# Photophysics of resveratrol derivatives for singlet oxygen formation†

**DOI:** 10.1039/d5cp00840a

**Published:** 2025-05-27

**Authors:** Mariana Yoshinaga, Josene M. Toldo, Willian R. Rocha, Mario Barbatti

**Affiliations:** a Laboratório de Estudos Computacionais em Sistemas Moleculares, eCsMo, Departamento de Química, ICEx, Universidade Federal de Minas Gerais Belo Horizonte Minas Gerais Brazil wrocha@ufmg.br; b University Claude Bernard Lyon 1, ENS de Lyon, CNRS, LCH, UMR 5182 69342 Lyon cedex 07 France; c Aix Marseille University, CNRS, ICR Marseille France mario.barbatti@univ-amu.fr; d Institut Universitaire de France 75231 Paris France

## Abstract

*trans*-Resveratrol, a naturally occurring antioxidant, undergoes significant photochemical transformations upon UV irradiation, producing photoisomers and derivatives such as *cis*-resveratrol, 2,4,6-trihydroxy-phenanthrene (THP), and resveratrone. Using quantum chemical methods, we investigated the photophysical properties of these species, including their absorption spectra, fluorescence, internal conversion (IC), and intersystem crossing (ISC) rates, to assess their potential for singlet oxygen generation. Our results indicate that while *trans*- and *cis*-resveratrol exhibit limited ISC, resveratrone and THP exhibit competitive ISC and fluorescence rates, making them suitable photosensitizers for singlet oxygen production at the same excitation energy as *trans*-resveratrol. However, THP is experimentally more abundant than resveratrone upon *trans*-resveratrol excitation and also demonstrates favorable ISC properties, suggesting that it plays a predominant role in singlet oxygen generation. These findings highlight the potential of resveratrone and THP in photodynamic applications, expanding the functional versatility of resveratrol-derived compounds.

## Abstract 2

Upon UV irradiation, *trans*-resveratrol forms *cis*-resveratrol, THP, and resveratrone. THP and resveratrone contribute most to singlet oxygen generation, although with low quantum yields.

## Graphical abstract

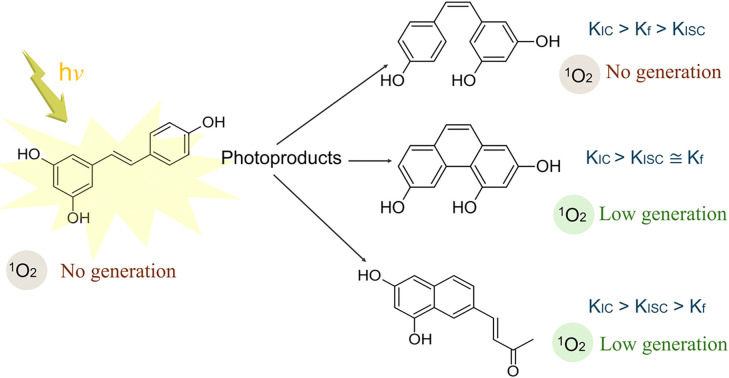

## 1 Introduction

Resveratrol is a naturally occurring compound that belongs to the class of phytoalexins, antimicrobial chemical substances produced by plants to combat infection by a pathogen.^1^ It is well-known for its antioxidant properties^2–6^ and is commonly found in grapes and peanuts.^7^ Although its most stable isomeric form is *trans*,^8^ upon exposure to ultraviolet (UV) radiation, resveratrol undergoes photoisomerization, originating its *cis* configuration.^9–11^ The experimentally determined molar yield of *cis*-resveratrol generated from *trans*-resveratrol excitation (in a hydroalcoholic solution exposed to solar irradiation) was approximately 60% and underwent significant degradation after 150 minutes.^12^ This interconversion was detailed in several computational and experimental works, demonstrating that the isomerization process is consistently observed for *trans*-resveratrol, in both solution and solid phases.^11,13^

**Fig. 1:**
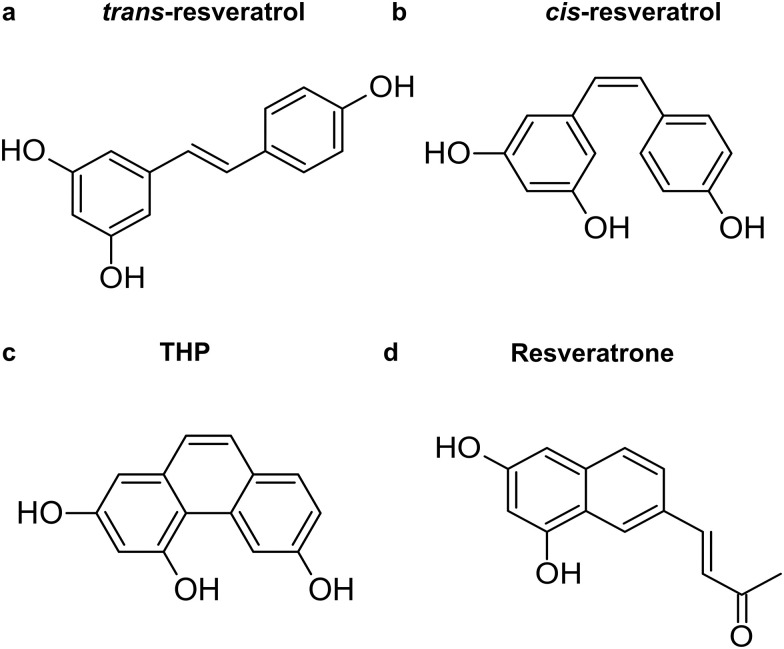
Structures of (a) *trans*-resveratrol and its byproducts (b) *cis*-resveratrol, (c) THP, and (d) resveratrone.

In addition to isomerization, photoexcited resveratrol can undergo reactions that result in byproducts (Fig. 1). Photoreactions are observed when *trans*-resveratrol is in a liquid environment but, unlike photoisomerization, they are not detected in the solid phase.^11^ An example of such a photoreaction is the cyclization of *trans*-resveratrol.^14,15^ After UV irradiation in water and ethanol solutions, the cyclized photoproduct 2,4,6-trihydroxy-phenanthrene (THP) was detected.^11,14,15^ Another photoproduct of *trans*-resveratrol excitation is (*E*)-4-(6,8-dihydroxy-naphthalen-2-yl)but-3-en-2-one, known as resveratrone.^12,16^ This product was proposed to be formed from the *cis* isomer, which can undergo a pericyclic ring closure, followed by a photoinduced 4e^−^ cyclization and by a series of tautomerization steps.

The experimental generation of THP and resveratrone by exposing hydroalcoholic solutions of *trans*- and *cis*-resveratrol to solar radiation shows that when *trans*-resveratrol is used as the precursor, the formation of photoproducts is higher (25.4% for THP and 11.7% for resveratrone)^12^ compared to their formation from the excitation of the *cis* isomer (16.5% for THP and 6.2% for resveratrone).^12^ This findings suggest that *trans*-resveratrol is a more effective precursor for these photoproducts under solar light exposure and that THP is produced in higher amounts than resveratrone, regardless of the isomer initially excited. The formation of THP is influenced by environmental factors, such as UV-light exposure conditions and solvent environment, which can be mitigated by using photo-protective agents in the formulations.^12^ Yet, Francioso *et al.* showed that THP exhibits cytotoxic and genotoxic effects, which were attributed to a possible ability to oxidize DNA.^14^

*trans*-Resveratrol is well known for its antioxidant capacity and ability to selectively quench singlet oxygen (^1^O_2_), a reactive species implicated in various oxidative stress-related diseases. The ability of quenching singlet oxygen is attributed to specific structural characteristics.^17–19^

While primarily recognized as an antioxidant, resveratrol's potential to generate singlet oxygen remains debated, although several studies have reported singlet oxygen generation upon photoexcitation.^20–24^ In this process, the excited molecule transfers energy to molecular oxygen (^3^O_2_), converting it into singlet oxygen (^1^O_2_), a key mechanism in photodynamic therapy (PDT). However, it's believed that it's not resveratrol that can generate singlet oxygen but certain of its derivatives. Fotiou *et al.*^20^ experimentally concluded that *trans*-resveratrol excitation under UV radiation can generate reactive oxygen species, such as ^1^O_2_ and peroxynitrite (ONOO^−^). However, their study does not clarify the structure of the compound directly responsible for the photosensitization.^20^

Zhao *et al.*^11^ proposed that *trans*-resveratrol, after excitation, undergoes cyclization and oxidation to form THP, which will then react with *trans*-resveratrol through a [4+2] cycloaddition. The generation of singlet oxygen by the THP compound was not experimentally confirmed by Zhao *et al.*^11^ but was assumed based on results observed for other phenanthrene-like compounds.^25^

Lagunes *et al.*^22^ attribute the formation of ^1^O_2_ and its subsequent reactions with various compounds present in wine, to changes in its taste and aroma. On the other hand, Monsour *et al.*^26^ demonstrated the formation of photoproducts and quenching of singlet oxygen but revealed that neither resveratrol nor its derivatives could act as photosensitizer for singlet oxygen production.^26^

**Scheme 1:**
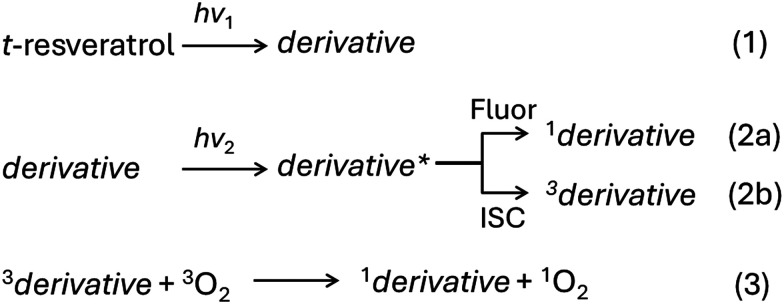
Relevant reactions to singlet oxygen generation after photoexcitation of *trans*-resveratrol. The derivatives investigated in this work are *trans*-resveratrol itself plus *cis*-resveratrol, THP, and resveratrone.

Understanding the ability of *trans*-resveratrol and its photoproducts to yield singlet oxygen is essential as it directly influences the applicability of these compounds for therapeutical purposes. The present study uses quantum chemistry methods to investigate the photophysical properties of *trans*-resveratrol and its derivatives *cis*-resveratrol, THP, and resveratrone to elucidate the potential of these molecules to yield singlet oxygen. We assess three aspects of the reaction sequence schematically illustrated in Scheme 1: (i) the absorption band overlap between *trans*-resveratrol precursor and the derivatives (*i.e.*, the difference between *hν*_1_ and *hν*_2_ in reactions (1) and (2)); (ii) the competition between fluorescence (reaction (2a)), internal convertion (IC), and intersystem crossing (ISC) (reaction (2b)); (iii) the energetic availability to excite a singlet oxygen state (reaction (3)). By exploring these properties, we can better understand the role of *trans*-resveratrol in singlet oxygen generation. The possibility of using *trans*-resveratrol as a photosensitizer to produce highly reactive oxygen species makes it attractive for photodynamic therapy, broadening its potential beyond its current use as an antioxidant.

## 2 Computational details

Geometry optimizations for the ground state (S_0_) was conducted using density functional theory (DFT)^27^ and the optimizations of excited singlet (S_1_) and lowest triplet (T) states were conducted using time-dependent density functional theory (TD-DFT) within the Tamm–Dancoff approximation (TDA).^28^ All calculations were performed using the ORCA 5.0.4 program.^29^ Geometry optimizations and frequency calculations were computed using CAM-B3LYP^30^ long-range exchange–correlation corrected functional in combination with the Ahlrichs full-electron Def2-TZVP basis set.^31^ Dispersion corrections were considered, including the D3 correction proposed by Grimme *et al.*^32^ with the Becke–Johnson (BJ)^33^ damping D3BJ. The chain-of-spheres approximation (COSX) was employed for the exchange term of the Fock matrix, using the auxiliary Def2/J basis set^34^ alongside the resolution-of-identity (RI)^35^ method for the Coulomb term. The solvent effects were accounted for using the CPCM implicit solvation model^36^ and with an explicit microsolvation (3 water molecules) in addition to CPCM. The latter will be referred to throughout the text as the explicit solvent.

Fluorescence and ISC rates were determined using TDA and the path integral approach proposed by de Souza *et al.*^37^ and implemented in ORCA. This approach provided reliable fluorescence, phosphorescence, and ISC rate constants predictions, as evidenced by several works.^37–40^

For fluorescence rates, the path integral approach starts from Fermi's golden rule^37^1
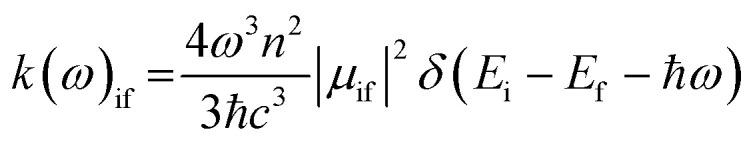
where, i and f correspond to the initial and final states, *μ* represents the transition dipole elements, and *ω* denotes the frequency of the emitted or absorbed photon. Then, the approach reformulates the Dirac delta function in the time domain, enabling the transition rate to be derived from the Fourier transform of a correlation function calculated using a path integral framework incorporating vibrational mode dynamics.

For ISC rates, the path integral approach is also based on Fermi's golden rule and quite similar to eqn (1) except for the frequency term. It is expressed as^37^2
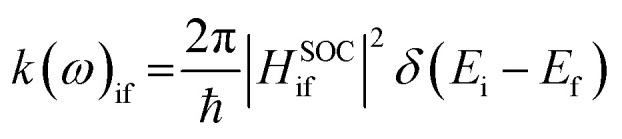
where the coupling |*H*^SOC^_if_|^2^ is given by the spin–orbit coupling matrix elements (SOCME) between the relevant states. The SOCME values were calculated using quasi-degenerate perturbation theory (QDPT),^41^ using TDA^42,43^ to describe the singlet–triplet interactions computed at the CAM-B3LYP/Def2-TZVP level.^30^ Energy gaps between the relevant singlet and triplet excited states were also computed at the same level of theory. The ISC rates were averaged over the three spin substates (*M*_s_ = −1, 0, and +1). The total ISC rate (*k*_ISC_) is given by the sum over all ISC rates from the S_1_ state to the *m*-th excited electronic triplet state energetically below.^44^ Fluorescence and intersystem crossing (ISC) rate constant calculations included Herzberg–Teller (HT) and Duschinsky rotation effects. Adiabatic Hessian model and a higher-level DFT integration grid (defgrid3) were employed in all cases. A Lorentzian broadening function was used throughout this work. All vibronic coupling calculations required Hessian matrices, which were consistently computed at the TDA/CAM-B3LYP/def2-TZVP level within the corresponding solvation models.

The internal conversion (IC) rates between the S_1_ and S_0_ states for THP and resveratrone were calculated using the excited state dynamics module of ORCA 6.0.0, employing the adiabatic Hessian model with the electron translation factor and computing the nonadiabatic coupling matrix elements at the TDA/CAM-B3LYP/Def2-TZVP level, considering explicit solvation model.

Due to computational cost, the explicit solvent model includes three water molecules forming the strongest hydrogen bonds with the solute. Bulk solvation and long-range interactions are captured *via* an implicit model. While increasing the number of explicit solvent molecules may affect absolute rate values, the comparative ISC and fluorescence trends discussed here are expected to remain valid. The position of these three molecules was determined through Monte Carlo (MC)^45,46^ simulations, employing standard Metropolis^47^ sampling protocols within the canonical NPT ensemble. The simulations were performed at a temperature of 25 °C in a cubic simulation box, where the volume was defined based on the experimental density of water and included 1000 solvent molecules. The complete simulation involves a thermalization stage of 100 000 MC steps and a production stage of 100 000 MC steps for averaging. All MC simulations were performed using the DICE program.^48^ The most relevant structures containing three hydrogen bonds were selected for further optimization with explicit solvation.

## 3 Results and discussion

### 3.1 Geometry optimizations

Upon the excitation of *trans*-resveratrol, one possible isomerization product is *cis*-resveratrol. As noted in the Introduction, the formation of cyclization products such as THP is well-documented, along with the photoreaction product resveratrone. The present study aims to clarify whether any compounds obtained after *trans*-resveratrol excitation can participate in singlet oxygen generation reactions. Thus, the photophysics of *trans*-resveratrol, *cis*-resveratrol, THP, and resveratrone was studied in implicit solvent and with three explicit water molecules plus implicit solvent, referred to as microsolvation. As discussed elsewhere,^49^ microsolvation is essential for describing excited state dynamics and the efficiency of photosensitizers in water, especially in cases where strong hydrogen bonds with the chromophore can be formed. Thus, these results should be considered our most accurate predictions.

**Fig. 2:**
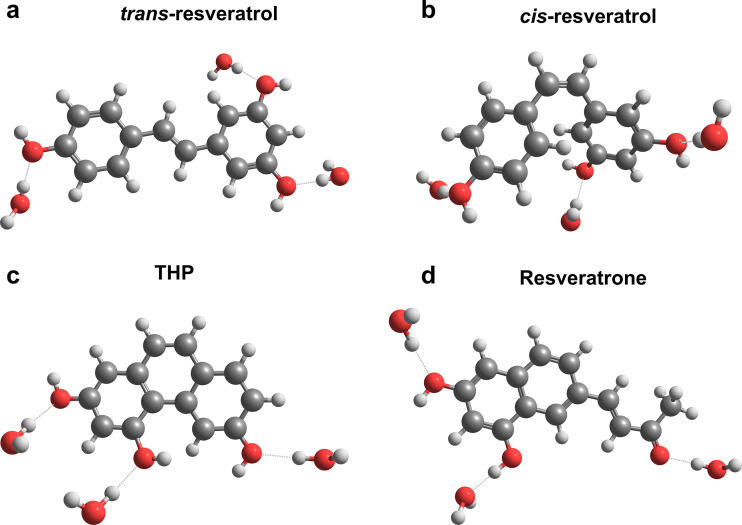
Optimized geometries of S_0_ minima including explicit + implicit water solvation for (a) *trans*-resveratrol, (b) *cis*-resveratrol, (c) THP, and (d) resveratrone.

The most favorable configurations of water molecules around the hydroxyl groups in each one of the molecules were extracted from MC simulations and further optimized at the DFT level for the ground state (Fig. 2) and TDA for the excited states.

### 3.2 Photophysical characterization

As we discussed in ref. 9, *trans*-resveratrol efficiently returns to the ground state *via* internal conversion near a conical intersection named twisted-pyramidalized (Tw-pyr). This pathway is barrierless and yields both *trans* and *cis* isomers. Indeed, it has been verified that isomerization to the *cis* isomer can occur through a conical intersection leading to the ground state *cis*-isomer. Alternatively, it could surpass a 0.64 eV barrier in the excited state, giving rise to the singlet excited *cis*-isomer. From there, a secondary pathway can generate a closed-ring derivative, through a cyclic conical intersection reached without an energy barrier. This closed-ring derivative could then undergo oxidation to form the THP compound.

**Fig. 3:**
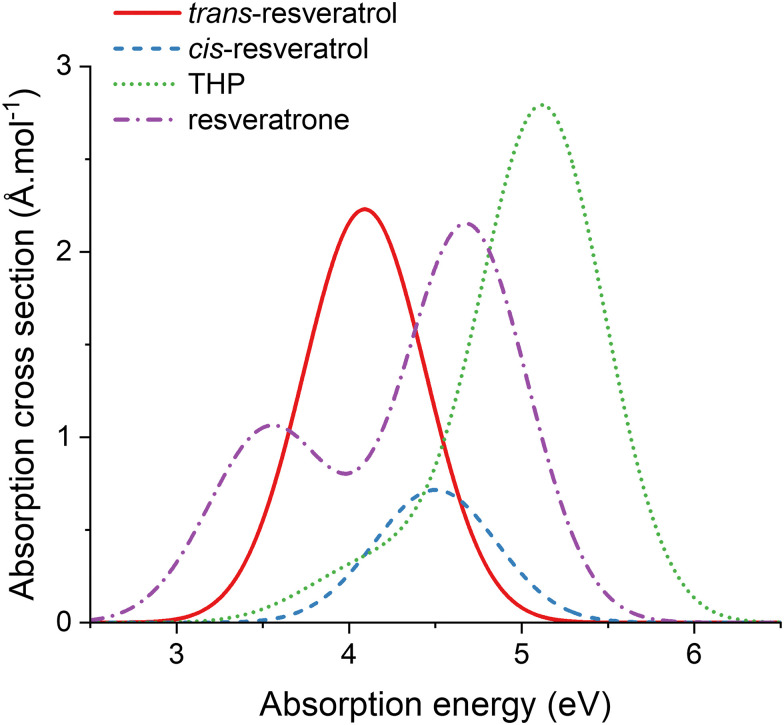
Vertical absorption spectrum convoluted using the main electronic transitions for *trans*-resveratrol, *cis*-resveratrol, THP and resveratrone.

Table 1 summarizes the vertical excitation of *trans*-resveratrol, *cis*-resveratrol, THP, and resveratrone in microsolvation. For each molecule, the excitations presented in the table were convoluted with Gaussian functions and are shown in Fig. 3 to better visualize the bands' overlap. The Gaussian convolution follows eqn (13) of ref. 50, where we adopted the same refractive index (1.33), Gaussian width (0.5 eV), and vertical-to-maximum band shift (0.1 eV) for all molecules.

**Table 1:** Vertical excitation energies (in eV and nm), oscillator strengths and main associated transitions for the lowest electronic singlet states of *trans*-resveratrol, *cis*-resveratrol, THP, and resveratrone calculated within the microsolvation model. The orbitals involved in the main transitions and their energies are shown in Fig. S1 (ESI)

	*E* (eV)	*λ* (nm)	*f*_osc._	Transition
*trans*-Resveratrol	4.19	295.9	1.354	S_0_ → S_1_
		306.0^a^		
*cis*-Resveratrol	4.60	269.8	0.435	S_0_ → S_1_
		285.0^a^		
THP	4.15	299.0	0.141	S_0_ → S_1_
	4.49	276.2	0.073	S_0_ → S_2_
	5.03	246.5	0.178	S_0_ → S_3_
	5.24	236.8	1.534	S_0_ → S_4_
		261.0^a^		
Resveratrone	3.64	340.4	0.635	S_0_ → S_1_
	4.20	294.9	0.013	S_0_ → S_2_
	4.24	292.7	0.0001	S_0_ → S_3_
	4.78	259.4	1.301	S_0_ → S_4_
		290.0^b^		

a Experimental wavelength with maximum absorbance in ethanol (ref. 11).

b Experimental wavelength with maximum absorbance in methanol (ref. 16).

To be suitable to use as a photosensitizer in phototherapy, we expect that the absorption band of the derivative significantly overlaps with that of the *trans*-resveratrol. This is because a single excitation energy is usually employed (*hν*_1_ = *hν*_2_ in Scheme 1) and then, it should excite both, *trans*-resveratrol and the derivative responsible for singlet oxygen generation.

*cis*-Resveratrol absorption occurs in a spectral region that overlaps with *trans*-resveratrol absorption (Fig. 3). The maximum absorption peak of THP, dominated by excitation into S_4_, is largely blue shifted when compared to *trans*- and *cis*- resveratrol. However, it still significantly overlaps with *trans*-resveratrol absorption thanks to the shoulder of excitations into S_1_ to S_3_. Resveratrone has two absorption peaks in the region of interest, one red-shifted (S_0_ → S_1_) and the other blue-shifted (S_0_ → S_4_). The combined tails of these bands cause a strong absorption in the same spectral absorption region of *trans*-resveratrol. Therefore, all three derivatives can be excited in single-energy setups, initially tuned to excite *trans*-resveratrol.

### 3.3 Fluorescence and ISC rates

A key step for the singlet oxygen formation is the ISC of the photosensitizer, populating the triplet states (Scheme 1, reaction (2b)).^51–54^ Therefore, it is essential to evaluate the ISC rates of the derivatives. After excitation to their bright states, the compounds would typically vibrationally relax to the S_1_ excited state, following Kasha's rule.^55^ From the S_1_ state minimum, fluorescence and intersystem crossing can compete (reactions (2a) and (2b)). To evaluate which compounds under study would be more prone for singlet oxygen generation upon excitation of *trans*-resveratrol, we calculated their fluorescence and ISC rates.

We computed S_1_ → T_*m*_ ISC rates for all triplet states *m*, whose energy at the T_*m*_ minimum was smaller than the S_1_ minimum energy, aiming at computational cost reduction. The ISC rates were computed using the path-integral formalism under the harmonic approximation, which treats vibrational effects at finite temperature. Nonetheless, we used strict adiabatic energy gaps to define accessible triplet states. This approach neglects the possibility that low-frequency vibrational motions could transiently bring otherwise inaccessible states into near resonance. Such vibronic effects may enhance the density of accessible states, particularly in flexible systems, and represent a limitation to the present approach.^56^

**Fig. 4:**
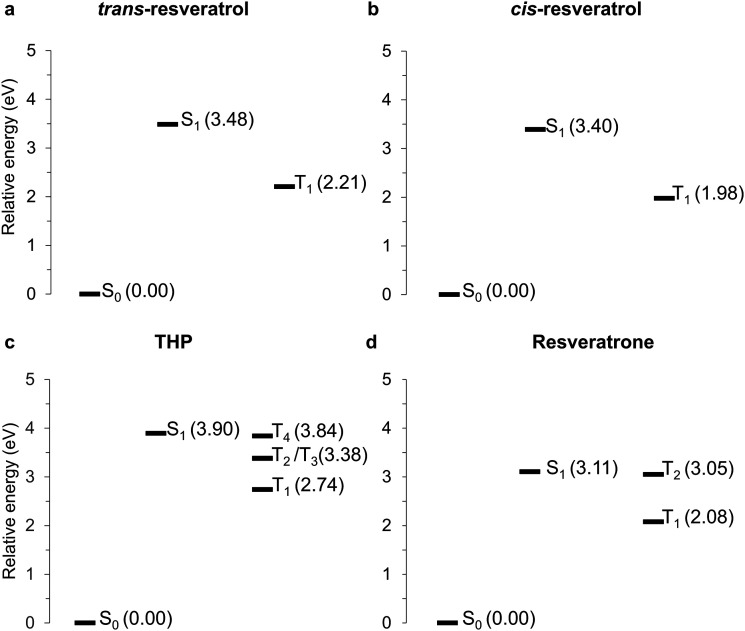
Schematic energy diagram (considering microsolvation) for (a) *trans*-resveratrol, (b) *cis*-resveratrol, (c) THP, and (d) resveratrone showing the energies of the singlet and triplet states at their respective optimized geometries. The energy values are relative to the S_0_ minimum.

The energies of each state at their respective optimized minima are listed in Table S1 in the ESI.†Fig. 4 shows the relative energies (in relation to the S_0_) of all excited states at their optimized geometries. Therefore, for *trans*- and *cis*-resveratrol, only ISC from S_1_ → T_1_ was computed, while for THP, we considered ISC from S_1_ to T_1_–T_4_ and for resveratrone, from S_1_ to T_1_ and T_2_. The relative energies of the compounds considering only the implicit solvent are presented in the ESI† (Fig. S2).

The calculated ISC rates in CPCM and explicit water are presented in Table 2. Looking at the explicit model calculations for THP, the transitions to the T_2_, T_3_, and T_4_ states exhibit practically equivalent intersystem crossing rates. In the case of resveratrone, the S_1_ → T_2_ transition has the most significant contribution to the ISC rate.

**Table 2:** ISC rates (in s^−1^) for *trans*-resveratrol, *cis*-resveratrol, THP, and resveratrone for all transitions with the triplet state minimum energy lower than the S_1_ minimum energy in the microsolvation model

		S_1_ → T_1_	S_1_ → T_2_	S_1_ → T_3_	S_1_ → T_4_
CPCM	*trans*-Resveratrol	2.99 × 10^5^	—	—	—
	*cis*-Resveratrol	1.83 × 10^1^	—	—	—
	THP	7.39 × 10^3^	1.13 × 10^6^	5.83 × 10^5^	1.22 × 10^6^
	Resveratrone	4.30 × 10^6^	1.82 × 10^7^	—	—
Explicit	*trans*-Resveratrol	2.85 × 10^1^	—	—	—
	*cis*-Resveratrol	3.90 × 10^6^	—	—	—
	THP	3.48 × 10^−9^	5.11 × 10^6^	6.89 × 10^6^	1.09 × 10^6^
	Resveratrone	3.33 × 10^4^	5.17 × 10^7^	—	—

In most cases, the calculated ISC rates in CPCM only and explicit solvent were equivalent. However, significant variations are observed in the ISC rate for the S_1_ → T_1_ employing these two solvation models. In the case of THP, where the ISC rate with microsolvation 12 orders of magnitude lower than in the implicit model, we found that the total reorganization energy in the explicitly solvated system was approximately 4000 times higher. Analyzing the vibrational modes that contribute significantly to this reorganization energy, we observed that they predominantly involve explicit solvent molecules. This suggests that the reorganization process required for intersystem crossing is more complex in the explicitly solvated environment. The presence of explicit water molecules introduces new interactions and vibrational modes that may modify vibronic coupling and ultimately reduce the efficiency of the ISC process. Such a strong dependence of ISC rates on explicit *vs.* implicit solvation has been previously reported in ref. 49. As mentioned, the explicit solvation results should be considered the most accurate.

Table 3 shows the fluorescence rate and the total ISC rate calculated from the sum^44^ of the individual ISC rates showed in Table 2. In all cases, ISC transitions occur between π–π* states, resulting in small spin–orbit coupling (SOC) values (below 0.1 cm^−1^, see Table S2 in the ESI†). These values are consistent with previous studies on related organic systems. For instance, singlet-triple SOC values between π–π* states in organic aromatic compounds composed exclusively of elements up to the third row of the periodic table calculated with ADC(2)^57^ and TDDFT^58^ have been reported to be similarly small.

**Table 3:** Total intersystem crossing and fluorescence rates for *trans*-resveratrol, *cis*-resveratrol, THP, and resveratrone in implicit solvent and microsolvation at 298 K

	*k* (s^−1^)	*trans*-Resveratrol	*cis*-Resveratrol	THP	Resveratrone
CPCM	Total ISC	2.99 × 10^5^	1.83 × 10^1^	2.94 × 10^6^	2.25 × 10^7^
	Fluorescence	2.60 × 10^8^	2.59 × 10^8^	2.00 × 10^8^	1.63 × 10^8^
Explicit	Total ISC	2.85 × 10^1^	3.90 × 10^6^	1.31 × 10^7^	5.17 × 10^7^
	Fluorescence	1.03 × 10^9^	2.04 × 10^9^	1.96 × 10^7^	6.29 × 10^6^

The ISC rate strongly depends on the energy gap between the states involved in the transition (see eqn (2)). Considering the results from explicit solvent systems, for the *trans* and *cis* resveratrol, the adiabatic (minimum-to-minimum) S_1_ → T_1_ energy gaps are 1.27 eV and 1.42 eV, respectively. In the case of THP, the adiabatic energy gap for S_1_ → T_2_ and S_1_ → T_3_ transitions is 0.52 eV and only 0.06 eV for the S_1_ → T_4_ transition. These low adiabatic energy gaps contribute to higher ISC rate of THP (1.31 × 10^7^ s^−1^) compared to those of the *trans*-resveratrol (2.85 × 10^1^ s^−1^) and *cis*-resveratrol (3.90 × 10^6^ s^−1^). A similar reasoning applies to resveratrone, where the ISC to the T_2_ state contributes most to the total ISC rate (5.17 × 10^7^ s^−1^), and has an adiabatic energy gap of only 0.06 eV.

The results shown in Table 3 are consistent with both experimental and theoretical data for similar compounds. The experimental fluorescence rate constant of *meta*-amino stilbene in acetonitrile solvent, for instance, is 5 × 10^7^ s^−1^, while the rate constant for nonradiative processes is 4 × 10^7^ s^−1^.^59^ For comparison, the experimentally determined ISC rate constant in solid solution for anthracene is 4.0 × 10^7^ s^−1^.^60^ Computational studies at ADC(2) level in gas phase agree with this ISC value (∼10^7^ s^−1^) and provide radiative rate constants also in the order of ∼10^7^.^57^ These computational results are also consistent with those obtained by Manian and collaborators.^61^

The explicit inclusion of hydrogen bonds between water and resveratrol derivatives through explicit solvation has a minor impact on the total ISC rates of THP and resveratrone. However, a dramatic effect is observed in *trans*- and *cis*-resveratrol. For fluorescence rates, explicit solvation has a small but still significant impact.

Nevertheless, the fluorescence rate values obtained for *cis*- and *trans*-resveratrol may contain inaccuracies due to significant geometric variations between the S_0_ and S_1_ states (RMSD greater than 0.25 Å). These structural changes considerably affect the Duschinsky displacement vector (*K⃑*), leading to large scalar product values (*K⃑*·*K⃑* > 7), which compromises the validity of the harmonic approximation for these systems. Such variations were not observed for THP or resveratrone, for which the calculated fluorescence rates are considered accurate within the limits of the employed methodology.

Internal conversion processes can also compete with ISC and fluorescence rates. For *trans* and *cis* resveratrol, internal conversion is the dominant deactivation pathway, as shown in our previous work. For THP and resveratrone, the internal conversion rates employing explicit solvent model were computed as 10^10^ and 10^8^ s^−1^, respectively.

Yet, comparing fluorescence and total ISC rates, we observed that the ISC process for *trans*- and *cis*-resveratrol is not competitive with fluorescence, being at least 1000 times slower for both implicit and explicit solvents. This result confirms that *trans*- and *cis*-resveratrol are not the compounds responsible for singlet oxygen generation after excitation of *trans*-resveratrol. The results for *cis*- and *trans*-resveratrol were included in this paper primarily for completeness, as our previous work (ref. 9) demonstrated that both compounds rapidly decay to the ground state *via* internal conversion, and therefore, low fluorescence and intersystem crossing rates were expected.

The total ISC rate for THP is 100 times smaller than the fluorescence rate when considering only the implicit solvent. However, it is about the same as the fluorescence rate when considering the explicit solvent. Thus, ISC may compete with fluorescence in THP.

In the case of resveratrone, the ISC rate is ten times smaller than the fluorescence rate, when considering only the implicit solvent. Still, it presents an ISC rate 8 times higher than the fluorescence rate when considering the explicit solvent, which indicates that resveratrone can have ISC and may overpower fluorescence.

The internal conversion rate calculated for THP was approximately 1000 times higher than the fluorescence and ISC rates. For resveratrone, the IC rate was about 100 times greater than the fluorescence rate and 10 times higher than the ISC rate. These results indicate that internal conversion is the predominant deactivation pathway for both compounds.

### 3.4 Oxygen singlet generation

Several key factors influence singlet oxygen generation. Among them, the intersystem crossing efficiency and the energetic balance between singlet and triplet states are recognized as primary determinants for singlet oxygen sensitization in organic molecules.^62^ Nevertheless, it is important to note that, additional factors such as the excitation energy of the photosensitizer, triplet state lifetime, energy transfer efficiency to molecular oxygen, possible charge recombination pathways, and the chemical environment can also critically affect singlet oxygen production.^63^ In this paper, our goal is to identify which photoproducts are most likely responsible for oxygen singlet generation based on their ability to populate triplet excited states. A complete mechanistic treatment of triplet energy transfer to molecular oxygen, including collisional and spin-exchange dynamics is beyond the scope of the present work.

Once ISC occurs, the compounds could transfer energy to molecular oxygen for the generation of singlet oxygen through a spin-exchange reaction^52^ (reaction (3) in Scheme 1). To verify this possibility, the T_1_ → S_0_ vertical energy gap at the T_1_ minimum of each compound (acting as a photosensitizer) was calculated (Table 4). They show only a slight dependence on the type of solvation treatment.

**Table 4:** T_1_ → S_0_ vertical energy gap at the T_1_ minimum for *trans*-resveratrol, *cis*-resveratrol, THP, and resveratrone, considering implicit solvent and microsolvated model

	T_1_ → S_0_ (eV) implicit solvent	T_1_ → S_0_ (eV) explicit solvent
*trans*-Resveratrol	1.60	1.68
*cis*-Resveratrol	0.30	0.35
THP	2.40	2.39
Resveratrone	1.77	1.75

Molecular oxygen ^3^O_2_ has a triplet ground state (^3^Σ_g_^−^) and experimental excitation energies of 0.97 eV and 1.63 eV to the lowest singlet excited states (^1^Δ_g_ and ^1^Σ_g_^+^), respectively, S_1_ and S_2_.^64^ Analyzing the vertical T_1_ → S_0_ energy gap (Table 4), we observe that THP and resveratrone have enough energy to excite the ^3^O_2_ to both singlet states. Although we have already discarded *cis*- and *trans*-resveratrol as photosensitizers, we can still extend this energy analysis to them. T_1_*trans*-resveratrol would have enough energy to excite the ^1^Δ_g_ state and maybe the ^1^Σ_g_^+^. On the other hand, T_1_*cis*-resveratrol would not have enough energy to excite neither.

## 4 Conclusions

We studied the photophysical properties of *trans*-resveratrol and its main photochemical products, *cis*-resveratrol, THP, and resveratrone, to elucidate the singlet oxygen generation following the precursor's excitation.

We evaluated the singlet oxygen production using three key indicators: (i) absorption band overlap between the derivative and the precursor (a significant overlap is expected in photodynamic therapy to enable secondary excitations); (ii) fluorescence and total ISC rates (ISC must be faster than fluorescence); (iii) T_1_–S_0_ energy gap at the T_1_ minimum (enough energy must be available to promote oxygen from triplet to the singlet states). A summary of the results for these three indicators is given in Table 5.

**Table 5:** Summary of the three criteria analyzed to determine the most probable compound responsible for singlet oxygen generation after *trans*-resveratrol photoexcitation: (i) Abs. overlap: can the compound be excited at the same energy as *trans*-resveratrol? (ii) Rates: is the ISC rate bigger than or at least competitive with the fluorescence rate? (iii) T_1_–S_0_ gap: does the molecule have enough energy to excite the lowest oxygen singlet state?

	Abs. overlap	Rates	T_1_–S_0_ gap
*trans*-Resveratrol	Yes	No	Yes
*cis*-Resveratrol	Yes	No	No
THP	Yes	Yes	Yes
Resveratrone	Yes	Yes	Yes

We observed that *cis*-resveratrol, THP, and resveratrone exhibit a reasonable absorption overlap with the absorption band of *trans*-resveratrol. Concerning the rates, the results showed that ISC is not competitive with fluorescence for *trans*- and *cis*-resveratrol since the latter occurs at least 1000 times faster. For THP, however, ISC competes with fluorescence and, for resveratrone, ISC dominates. These are favorable indicators for the formation of singlet oxygen by the photochemical products generated after *trans*-resveratrol excitation.

Analyzing the T_1_ → S_0_ gap, we observed that THP and resveratrone have enough energy to excite molecular oxygen to the ^1^Δ_g_ and ^1^Σ_g_^+^ singlet states. *trans*-Resveratrol could excite the ^1^Δ_g_ state and maybe the ^1^Σ_g_^+^. *cis*-Resveratrol would not have enough energy to excite any oxygen singlet state.

The higher values of IC rates compared to ISC rates in the explicit solvent model for THP and resveratrone suggest that only a small fraction of the excited molecules undergo intersystem crossing. Considering that internal conversion and ISC are competitive but not mutually exclusive processes, THP and resveratrone can act as photosensitizers, although probably with low singlet oxygen yields.

Based on their distinct photophysical properties, THP and resveratrone could be potential photosensitizers for applications in photodynamic therapy and controlled oxidative processes. Nevertheless, structural modifications to resveratrol, such as incorporating heavier atoms to enhance spin–orbit coupling or modifications aimed at reducing internal conversion rates, could be explored to improve the efficiency of singlet oxygen production.

Future investigations should validate their photosensitizing efficiency under biologically relevant conditions and further examine the effects of solvent environment, molecular aggregation, and specific intermolecular interactions. A deeper understanding of these factors will be essential for optimizing the application of resveratrol derivatives in photochemical and biomedical contexts.

## Author contributions

Supervision: JMT, MB; project administration: MB; funding acquisition: WR, MB; methodology: JMT, MB; analysis: MY; investigation: MY; visualization: MY; writing – original draft: MY; writing – review & editing: MY, JMT, WR, MB.

## Conflicts of interest

The authors declare no conflict of interest.

## Supplementary Material

CP-027-D5CP00840A-s001

## Data Availability

The data supporting this article have been included in the ESI.†
