# Supplementary material for: Photophysics of resveratrol derivatives for singlet oxygen formation
Source: Phys Chem Chem Phys. 2025 May 27;27(23):12560–8. doi: 10.1039/d5cp00840a (PMC12132032; doi:10.1039/d5cp00840a)
Supplement: CP-027-D5CP00840A-s001 [file CP-027-D5CP00840A-s001.pdf]

Supplementary Information (SI) for Physical Chemistry Chemical Physics.
This journal is © the Owner Societies 2025

Supplementary information

Photophysics of Resveratrol Derivatives for Singlet Oxygen Formation

Mariana Yoshinaga,[1] Josene M. Toldo,[2,3] Willian R. Rocha,[1]* Mario Barbatti[3,4]*

[1] Laboratório de Estudos Computacionais em Sistemas Moleculares, eCsMo, Departamento de Química, ICEx, Universidade Federal de Minas Gerais, Belo Horizonte, Minas Gerais, Brazil
[2] University Claude Bernard Lyon 1, ENS de Lyon, CNRS, LCH, UMR 5182, 69342, Lyon cedex 07, France
[3]Aix Marseille University, CNRS, ICR, Marseille, France
[4] Institut Universitaire de France, 75231 Paris, France

*mario.barbatti@univ-amu.fr ; wrocha@ufmg.br

Table of Contents

S1. TDA-DFT calculations 2
S1. Cartesian coordinates 6

S1. TDA-DFT calculations

Table S1. Energies (in hartree) of the S_0, S_1, and T_m states for *trans*-resveratrol, *cis*-resveratrol, THP, and resveratrone, obtained using TDA-CAM-B3LYP/Def2-TZVP at their respective optimized geometries.

		trans-resveratrol	*cis*-resveratrol	THP	resveratrone
CPCM	S_0	-766.346	-766.339	-765.170	-766.354
	S_1	-766.217	-766.214	-765.025	-766.235
	T_1	-766.265	-766.266	-765.069	-766.276
	T_2	-766.215	-766.207	-765.044	-766.24
	T_3	-	-	-765.044	-766.209
	T_4	-	-	-765.025	-
Explicit	S_0	-995.703	-995.696	-994.541	-995.718
	S_1	-995.574	-995.571	-994.397	-995.603
	T_1	-995.621	-995.623	-994.439	-995.641
	T_2	-995.570	-995.560	-994.416	-995.605
	T_3	-	-	-994.415	-995.587
	T_4	-	-	-994.399	-

Table S2. Spin-orbit coupling values (cm^{-1}) for *trans*-, *cis*-, THP, and resveratrone for all transitions with the triplet state minimum energy lower than the S_1 minimum energy.

	$S_1 \rightarrow T_1$	$S_1 \rightarrow T_2$	$S_1 \rightarrow T_3$	$S_1 \rightarrow T_4$
CPCM				
trans-resveratrol	0.02	-	-	-
cis-resveratrol	0.00	-	-	-
THP	0.02	0.09	0.08	0.09
Resveratrone	0.02	0.10	-	-
Explicit				
trans-resveratrol	0.01	-	-	-
cis-resveratrol	0.10	-	-	-
THP	0.01	0.05	0.09	0.04
Resveratrone	0.02	0.09	-	-

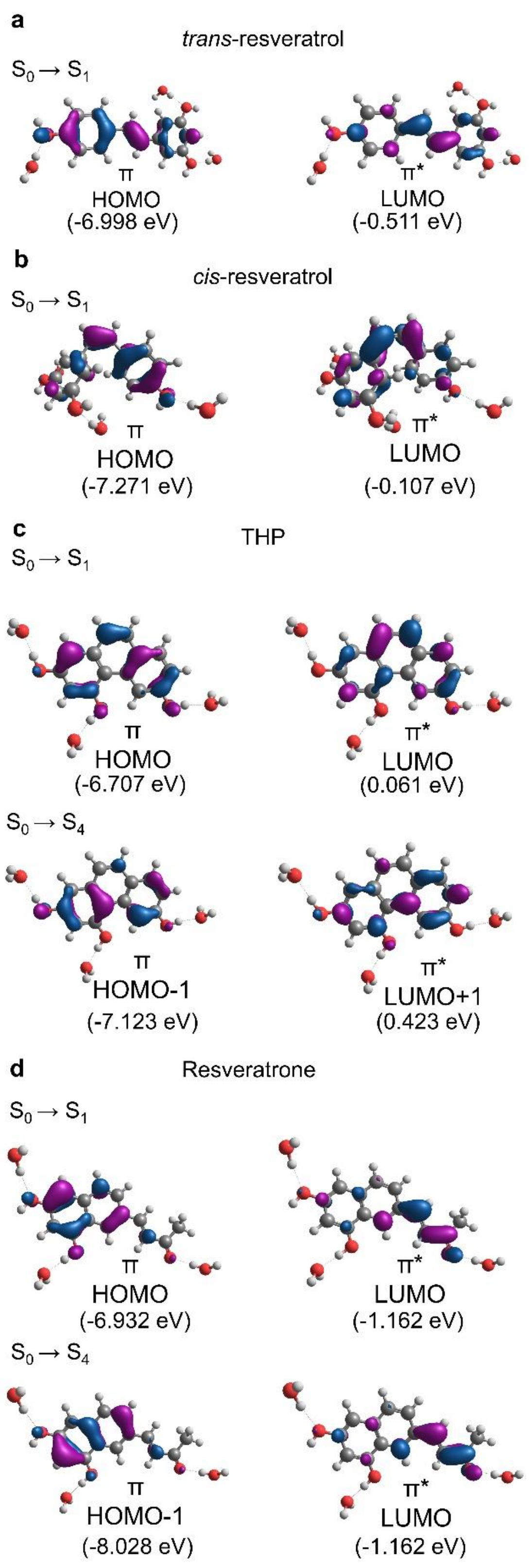

Figure S1. Kohn Sham orbitals involved in the main transitions of (a) trans-resveratrol, (b) cis-resveratrol, (c) THP, and (d) resveratrone computed at the TDA-CAM-B3LYP/Def2-TZVP in CPCM(water).

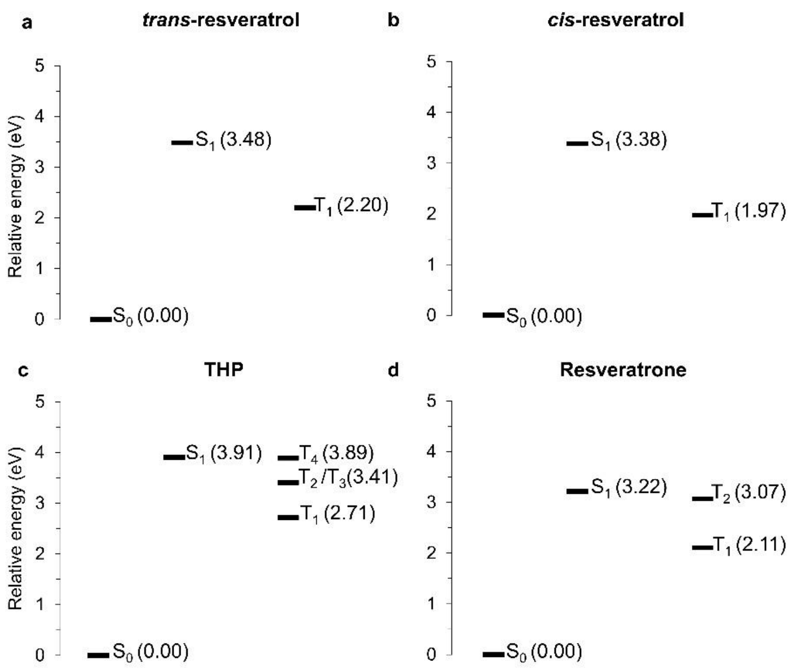

Figure S2. Schematic energy diagram (considering the implicit solvation) for (a) trans-resveratrol, (b) cis-resveratrol, (c) THP, and (d) resveratrone showing the relative energies of the singlet and triplet states at their respective optimized geometries. The energy differences are given taking the S_0 as reference.

S1. Cartesian coordinates

Cartesian coordinates in angstroms of the geometries optimized in implicit solvent at CAM-B3LYP/Def2-TZVP/cpcm(water) level

Trans-resveratrol

- **S_0**

O	-2.660495	5.016352	-3.118533
H	-3.334933	5.561370	-2.694033
C	0.013610	-0.048934	0.004781
H	0.519754	-0.048217	-0.954042
C	-0.910380	0.878309	0.257805
H	-1.410949	0.875601	1.220154
C	-1.347389	1.944900	-0.642187
C	-0.838074	2.125698	-1.931384
H	-0.079148	1.456950	-2.314390
H	-0.882245	3.279634	-3.737943
C	-1.282674	3.148055	-2.741427
C	-2.257717	4.026356	-2.280912
C	-2.779547	3.868627	-1.006719
C	-2.323643	2.837508	-0.204619
H	-2.736300	2.721194	0.789954
C	1.903791	-2.994317	1.299003
C	1.454664	-1.971322	0.478251
C	1.360977	-3.182351	2.559168
H	1.891177	-1.840140	-0.504465
H	1.718334	-3.984555	3.192152
C	0.446026	-1.113462	0.914412
C	0.355608	-2.326452	2.986463
O	-0.217849	-2.463615	4.211680
C	-0.104375	-1.299010	2.180767
H	-0.890000	-0.659643	2.556738
O	2.885161	-3.850567	0.911408
H	3.189175	-3.628950	0.022369
H	0.173461	-3.208259	4.685046
H	-3.539184	4.551750	-0.646343

- **S_1**

O	-2.614910	4.985217	-3.102888
H	-3.303303	5.534774	-2.703060
C	0.030529	-0.066849	-0.006469
H	0.517366	-0.029663	-0.972570
C	-0.963583	0.899202	0.280398
H	-1.460743	0.861546	1.240970
C	-1.363532	1.913555	-0.584059
C	-0.808580	2.108731	-1.892804
H	-0.036427	1.445645	-2.253868

H	-0.814839	3.268219	-3.687081
C	-1.237860	3.125938	-2.701077
C	-2.234665	4.003845	-2.265902
C	-2.805015	3.844850	-0.991272
C	-2.382296	2.834282	-0.180409
H	-2.822534	2.714264	0.801439
C	1.897220	-2.988274	1.261405
C	1.468493	-1.987478	0.431983
C	1.352901	-3.156345	2.542278
H	1.902513	-1.875842	-0.554446
H	1.706996	-3.954779	3.181739
C	0.448288	-1.080134	0.854899
C	0.355830	-2.279555	2.966271
O	-0.203148	-2.405885	4.201634
C	-0.100194	-1.260252	2.162773
H	-0.874232	-0.609228	2.540679
O	2.867101	-3.874355	0.903760
H	3.181152	-3.680221	0.011611
H	0.191828	-3.149764	4.672800
H	-3.577519	4.531061	-0.665578

- **T_1**

O	-6.029411	-1.665913	-0.407780
H	-6.635258	-0.929602	-0.562134
C	0.434633	-0.838316	-0.215574
H	0.316105	-1.899880	-0.390564
C	-0.722370	0.017638	-0.378889
H	-0.560483	1.078267	-0.515918
C	-2.044391	-0.424506	-0.383888
C	-2.422901	-1.784431	-0.182143
H	-1.662037	-2.531874	-0.011678
H	-4.013571	-3.205791	-0.039396
C	-3.739138	-2.170314	-0.193259
C	-4.746809	-1.230985	-0.403758
C	-4.415239	0.115419	-0.601158
C	-3.105346	0.504969	-0.591575
H	-2.855508	1.547063	-0.745800
C	4.037789	-0.935457	0.581113
C	2.775367	-1.342455	0.230205
C	4.314422	0.406977	0.855368
H	2.579565	-2.386411	0.018236
H	5.317711	0.709828	1.126410
C	1.715103	-0.399790	0.143147
C	3.284033	1.335938	0.768611
O	3.504035	2.654144	1.023633
C	2.003452	0.959848	0.422352
H	1.235991	1.717102	0.369585

O	5.084913	-1.798846	0.679026
H	4.795591	-2.695423	0.468210
H	4.430367	2.799565	1.251196
H	-5.204540	0.840047	-0.762689

Cis-resveratrol

- **S_0**

O	-1.043643	4.914732	-1.367135
H	-0.093858	5.064181	-1.454322
C	-1.672737	-1.549147	-0.348175
H	-2.090711	-2.543482	-0.465336
C	-2.316463	-0.549407	-0.950619
H	-3.247196	-0.803574	-1.448095
C	-1.937570	0.867997	-1.053242
C	-0.619564	1.285907	-1.222881
H	0.172084	0.552024	-1.278510
H	0.729519	2.935267	-1.467902
C	-0.299879	2.625582	-1.330793
C	-1.303401	3.583853	-1.271583
C	-2.625275	3.190213	-1.125480
C	-2.930427	1.845697	-1.029789
H	-3.966045	1.545993	-0.924837
C	1.655112	-2.462414	1.104999
C	0.504580	-2.489094	0.329401
C	1.858868	-1.461522	2.039828
H	0.367641	-3.285271	-0.390155
H	2.753105	-1.441037	2.650032
C	-0.451652	-1.492193	0.476284
C	0.886657	-0.483704	2.196421
O	1.118053	0.471204	3.136293
C	-0.263922	-0.492290	1.426923
H	-1.009660	0.278404	1.568858
O	2.563068	-3.455170	0.910121
H	3.314681	-3.345991	1.505870
H	0.384854	1.098835	3.161114
H	-3.403014	3.941664	-1.094690

S1

O	-1.076573	5.022799	-1.121999
H	-0.125791	5.197047	-1.090142
C	-1.470281	-1.482004	-0.647171
H	-1.672324	-2.396540	-1.198768
C	-2.332802	-0.381921	-0.891159
H	-3.370549	-0.609820	-1.121889
C	-1.971068	0.962558	-0.961606
C	-0.610105	1.411055	-0.964188
H	0.185513	0.683144	-0.973117

H	0.727433	3.078551	-1.028392
C	-0.305312	2.749602	-1.018688
C	-1.321095	3.699387	-1.080485
C	-2.667411	3.294875	-1.120259
C	-2.978489	1.969947	-1.074469
H	-4.015548	1.659390	-1.099934
C	1.496191	-2.680391	1.200411
C	0.457430	-2.645956	0.309339
C	1.698199	-1.649000	2.130106
H	0.339266	-3.446113	-0.409378
H	2.524676	-1.682950	2.828678
C	-0.439191	-1.538319	0.292526
C	0.804472	-0.586579	2.154745
O	1.015426	0.369215	3.101082
C	-0.254158	-0.514843	1.273760
H	-0.974158	0.284453	1.369188
O	2.340849	-3.747911	1.158654
H	3.026613	-3.660605	1.831852
H	0.357856	1.070708	3.014116
H	-3.438210	4.051040	-1.187343

- **T_1**

O	-1.283473	5.030642	-1.743330
H	-0.347277	5.205932	-1.900628
C	-1.323976	-1.424874	-0.554595
H	-1.426492	-2.248009	-1.256314
C	-2.290909	-0.342047	-0.632882
H	-3.337519	-0.597845	-0.497458
C	-1.998868	0.999459	-0.951857
C	-0.693491	1.458743	-1.237981
H	0.126925	0.755404	-1.240547
H	0.567241	3.120520	-1.716927
C	-0.441624	2.786202	-1.505359
C	-1.480801	3.709566	-1.500920
C	-2.783982	3.287162	-1.240672
C	-3.034151	1.965179	-0.975097
H	-4.047302	1.645412	-0.766128
C	1.676018	-2.685336	1.210068
C	0.634426	-2.594225	0.310598
C	1.841753	-1.736253	2.213303
H	0.530181	-3.340554	-0.465533
H	2.661697	-1.806113	2.917065
C	-0.279954	-1.524787	0.393118
C	0.932646	-0.687880	2.306649
O	1.134061	0.205734	3.311854
C	-0.116329	-0.569567	1.418730
H	-0.809288	0.255858	1.509487

O	2.532978	-3.733801	1.079172
H	3.217107	-3.697087	1.759151
H	0.457730	0.894194	3.285318
H	-3.584122	4.015625	-1.245681

THP

- **S_0**

O	3.972814	-1.885894	-0.000001
H	4.903026	-1.618656	-0.000001
C	-0.775596	2.394200	0.000001
H	-1.431833	3.266975	0.000001
C	0.568694	2.537582	0.000001
H	1.023641	3.531054	0.000001
C	1.434926	1.395268	0.000000
C	0.890760	0.077910	0.000000
H	1.447911	-2.024705	0.000000
C	1.802869	-1.001171	0.000000
C	3.172765	-0.794920	0.000000
C	3.700906	0.508516	-0.000001
C	2.833116	1.576419	0.000000
H	3.226195	2.595863	0.000000
C	-3.415184	-0.222462	0.000000
C	-2.790541	1.008783	0.000000
C	-2.638092	-1.389541	0.000000
H	-3.382525	1.927020	0.000000
H	-3.138410	-2.360263	0.000000
C	-1.383868	1.090449	0.000000
C	-1.255325	-1.325729	0.000001
O	-0.546140	-2.474919	0.000001
C	-0.564533	-0.074639	0.000001
O	-4.755008	-0.375789	-0.000001
H	-5.189888	0.489394	-0.000001
H	-1.145974	-3.234900	0.000001
H	4.783321	0.659657	-0.000001

- **S_1**

O	3.960043	-1.863955	0.087582
H	4.893059	-1.612631	0.115622
C	-0.799594	2.367008	-0.024069
H	-1.442528	3.238028	-0.039633
C	0.586775	2.528384	0.021082
H	1.020249	3.518974	0.040709
C	1.434194	1.417796	0.040242
C	0.882749	0.065912	0.014645
H	1.465589	-2.009112	0.014974
C	1.796465	-0.987732	0.033203

C	3.171936	-0.766863	0.074729
C	3.706754	0.524448	0.101271
C	2.841322	1.591621	0.084000
H	3.227152	2.602520	0.103176
C	-3.405189	-0.244802	-0.121085
C	-2.792604	0.992058	-0.095840
C	-2.638956	-1.408237	-0.100300
H	-3.395597	1.892724	-0.111870
H	-3.118921	-2.377306	-0.118728
C	-1.383429	1.107370	-0.049438
C	-1.245501	-1.316369	-0.054390
O	-0.546689	-2.466646	-0.033914
C	-0.554215	-0.086285	-0.028922
O	-4.749478	-0.393066	-0.166295
H	-5.185056	0.469207	-0.176912
H	-1.147300	-3.223628	-0.051278
H	4.779581	0.667582	0.134012

- **T_1**

O	3.960423	-1.873708	0.091080
H	4.896008	-1.633978	0.118437
C	-0.817454	2.355253	-0.025198
H	-1.458686	3.226191	-0.040234
C	0.607109	2.523653	0.020191
H	1.030489	3.517820	0.039041
C	1.439648	1.422370	0.039322
C	0.899828	0.080969	0.015037
H	1.455103	-1.987980	0.017842
C	1.794573	-0.968463	0.034905
C	3.186808	-0.760220	0.076967
C	3.716608	0.522976	0.101641
C	2.856909	1.589848	0.083153
H	3.246829	2.599056	0.101516
C	-3.415676	-0.250125	-0.120407
C	-2.808327	0.986743	-0.095142
C	-2.644710	-1.395728	-0.101179
H	-3.405130	1.890528	-0.109621
H	-3.118189	-2.368475	-0.120387
C	-1.393236	1.094195	-0.050100
C	-1.237388	-1.302105	-0.056011
O	-0.541257	-2.467001	-0.038473
C	-0.568565	-0.085304	-0.030298
O	-4.764068	-0.402396	-0.164255
H	-5.197719	0.460162	-0.173735
H	-1.148337	-3.217113	-0.055965

H	4.789213	0.669829	0.134448

- **T_2**

O	3.971612	-1.869553	0.089718
H	4.898023	-1.598075	0.116969
C	-0.790814	2.393451	-0.022299
H	-1.443671	3.257549	-0.037397
C	0.559786	2.548617	0.020624
H	1.004598	3.533763	0.040553
C	1.431589	1.411891	0.039504
C	0.869106	0.063373	0.014533
H	1.473292	-2.028079	0.017455
C	1.804408	-1.007962	0.034663
C	3.162464	-0.780254	0.075035
C	3.694249	0.510910	0.099689
C	2.805096	1.589956	0.081054
H	3.193616	2.599261	0.099105
C	-3.393316	-0.242410	-0.121531
C	-2.766392	1.002576	-0.094305
C	-2.626450	-1.416686	-0.100975
H	-3.370954	1.902974	-0.109688
H	-3.117459	-2.380044	-0.119541
C	-1.386044	1.109060	-0.048526
C	-1.255956	-1.339456	-0.055160
O	-0.546737	-2.475767	-0.033793
C	-0.537882	-0.081525	-0.028004
O	-4.731892	-0.380294	-0.167108
H	-5.167661	0.482820	-0.177153
H	-1.136828	-3.242169	-0.052447
H	4.765021	0.663070	0.131596

- T_3

O	3.967406	-1.870799	0.012962
H	4.894601	-1.602561	0.041337
C	-0.792438	2.394404	-0.000043
H	-1.445852	3.257905	-0.019001
C	0.558412	2.550859	0.058925
H	1.001960	3.536622	0.091214
C	1.430529	1.414347	0.062087
C	0.867858	0.065693	0.017981
H	1.469803	-2.025290	-0.055666
C	1.801677	-1.006169	-0.005758
C	3.159949	-0.780633	0.035178
C	3.691970	0.508832	0.094773
C	2.803890	1.589871	0.102973
H	3.193704	2.598540	0.139128
C	-3.388242	-0.245860	-0.155805
C	-2.763284	1.000441	-0.120428
C	-2.623373	-1.419118	-0.089747

H	-3.368966	1.898677	-0.157794
H	-3.113559	-2.383135	-0.086602
C	-1.386007	1.110740	-0.039009
C	-1.254968	-1.337655	-0.007509
O	-0.548261	-2.470631	0.086608
C	-0.537711	-0.079368	-0.004836
O	-4.724193	-0.382887	-0.240619
H	-5.156841	0.480852	-0.276803
H	-1.140314	-3.235624	0.081916
H	4.763056	0.658944	0.127112

- T_4

O	3.987520	-1.862460	0.094914
H	4.916276	-1.597831	0.123014
C	-0.777529	2.379136	-0.024844
H	-1.428550	3.243185	-0.039975
C	0.566505	2.528108	0.017175
H	1.011134	3.514968	0.036266
C	1.436494	1.395085	0.037663
C	0.898840	0.050436	0.014018
H	1.465180	-2.018161	0.019602
C	1.797935	-0.996074	0.035697
C	3.183494	-0.770426	0.078480
C	3.711953	0.544199	0.102531
C	2.845945	1.589600	0.082010
H	3.220638	2.605064	0.099469
C	-3.419031	-0.215491	-0.119036
C	-2.802956	0.995167	-0.094375
C	-2.645332	-1.397711	-0.101480
H	-3.384263	1.909037	-0.107683
H	-3.136217	-2.361460	-0.120402
C	-1.381003	1.079345	-0.050321
C	-1.234741	-1.321401	-0.058246
O	-0.552139	-2.493450	-0.043874
C	-0.561183	-0.106058	-0.031420
O	-4.763549	-0.390219	-0.160713
H	-5.211596	0.465638	-0.168824
H	-1.166491	-3.237725	-0.063266
H	4.783474	0.696501	0.136194

Resveratrone

- **S_0**

H	-0.772004	-0.055552	-3.471820
C	-0.614040	0.462130	-2.535528
C	-0.684375	1.826031	-2.484836
C	-0.480489	2.524764	-1.276975
C	-0.208699	1.836117	-0.130679

C	-0.129973	0.414516	-0.132132
C	-0.336638	-0.263197	-1.361020
C	-0.254501	-1.675600	-1.357375
C	0.010987	-2.359122	-0.210673
C	0.214267	-1.686776	1.019566
C	0.141654	-0.312966	1.036105
H	-0.539644	3.607466	-1.263347
H	0.292268	0.233351	1.955623
H	0.069181	-3.440445	-0.228421
H	-0.407935	-2.206922	-2.287914
C	0.487959	-2.486415	2.207581
H	0.516818	-3.555974	2.030423
C	0.696774	-2.042944	3.450460
H	0.679375	-0.986574	3.689927
C	0.962909	-2.915597	4.596399
C	1.014479	-4.406402	4.409804
H	1.215087	-4.879772	5.366956
H	0.069480	-4.776727	4.011105
H	1.796380	-4.676640	3.699224
O	-0.955616	2.502160	-3.627915
H	-0.983919	3.452658	-3.462982
O	-0.001420	2.451081	1.055217
H	-0.062145	3.409547	0.954228
O	1.142048	-2.421748	5.699928

- **S_1**

H	-0.810939	-0.040327	-3.437736
C	-0.631162	0.455370	-2.493385
C	-0.685277	1.860735	-2.445239
C	-0.458359	2.542153	-1.256677
C	-0.178510	1.830381	-0.110472
C	-0.118726	0.390680	-0.110081
C	-0.354869	-0.270964	-1.356379
C	-0.293649	-1.705360	-1.373243
C	-0.026099	-2.405960	-0.247219
C	0.205745	-1.764411	1.004083
C	0.151944	-0.328868	1.028895
H	-0.499300	3.625052	-1.228586
H	0.326262	0.196835	1.954377
H	0.014665	-3.487476	-0.280988
H	-0.467987	-2.213970	-2.312364
C	0.470695	-2.538991	2.143287
H	0.488261	-3.611057	1.991796
C	0.702901	-2.042794	3.420870
H	0.687347	-0.974671	3.601486
C	0.970625	-2.843166	4.571648
C	1.018155	-4.346006	4.432827

H	1.238243	-4.789179	5.400990
H	0.066028	-4.737702	4.069350
H	1.783848	-4.652002	3.717349
O	-0.962236	2.489691	-3.592697
H	-0.984774	3.449624	-3.472523
O	0.052178	2.423397	1.058380
H	0.006407	3.387560	0.981630
O	1.166854	-2.328122	5.691552

- **T_1**

H	-0.826354	-0.045636	-3.437032
C	-0.641493	0.457539	-2.497061
C	-0.687489	1.852832	-2.448750
C	-0.452588	2.532146	-1.258451
C	-0.172618	1.821079	-0.111545
C	-0.120020	0.397803	-0.115076
C	-0.363384	-0.262579	-1.355076
C	-0.307434	-1.705214	-1.369280
C	-0.039105	-2.411058	-0.255045
C	0.203799	-1.770546	1.007410
C	0.155464	-0.333433	1.032408
H	-0.488761	3.615452	-1.231079
H	0.335403	0.193683	1.954665
H	-0.002905	-3.492644	-0.290592
H	-0.488946	-2.210417	-2.309572
C	0.467227	-2.546415	2.127088
H	0.480240	-3.618519	1.983111
C	0.711048	-2.041738	3.419067
H	0.701539	-0.975466	3.600425
C	0.980402	-2.844378	4.574018
C	1.022309	-4.344342	4.446671
H	1.240146	-4.783148	5.416773
H	0.068415	-4.729302	4.082764
H	1.787458	-4.651124	3.731868
O	-0.966495	2.500248	-3.598697
H	-0.979597	3.456413	-3.460331
O	0.064226	2.428357	1.066116
H	0.018975	3.388933	0.972093
O	1.178808	-2.308078	5.674040

- **T_2**

H	-0.805946	-0.008294	-3.480584
C	-0.630137	0.484226	-2.534161
C	-0.681643	1.875460	-2.465189
C	-0.461535	2.546914	-1.277546
C	-0.180820	1.802590	-0.111439
C	-0.121946	0.391273	-0.124972

C	-0.349598	-0.284689	-1.365646
C	-0.288832	-1.671803	-1.388357
C	-0.015003	-2.408706	-0.230782
C	0.203736	-1.753998	0.987582
C	0.148267	-0.355343	1.021727
H	-0.502741	3.628356	-1.234050
H	0.317864	0.170954	1.948636
H	0.026397	-3.488023	-0.271943
H	-0.458490	-2.187551	-2.324944
C	0.478387	-2.534432	2.169345
H	0.516250	-3.606040	2.017234
C	0.684473	-2.049545	3.442935
H	0.649732	-0.986869	3.646482
C	0.954477	-2.872984	4.598100
C	1.029638	-4.367765	4.452643
H	1.236246	-4.813779	5.421716
H	0.091606	-4.766599	4.063534
H	1.816215	-4.647922	3.750305
O	-0.956160	2.529110	-3.614614
H	-0.975162	3.483768	-3.467796
O	0.041027	2.422322	1.063000
H	-0.010549	3.381508	0.959691
O	1.122518	-2.341688	5.700023

Cartesian coordinates in angstroms of the geometries optimized in explict solvent at CAM-B3LYP/Def2-TZVP/cpcm(water) level.

Trans-resveratrol

- **S_0**

O	-2.766247	5.228088	-2.108728
H	-3.466297	5.618092	-1.569621
C	0.396212	-0.152974	-0.204216
H	0.873216	0.095028	-1.145640
C	-0.591208	0.616227	0.254690
H	-1.067176	0.360192	1.195101
C	-1.135982	1.813977	-0.383647
C	-0.668510	2.322579	-1.598513
H	0.139090	1.827681	-2.120241
H	-0.845827	3.844625	-3.098692
C	-1.216934	3.455468	-2.160009
C	-2.254671	4.113721	-1.512345
C	-2.737468	3.633610	-0.307283
C	-2.177534	2.494558	0.243511
H	-2.558482	2.122579	1.186532
C	2.484927	-3.189678	0.368213
C	1.947360	-2.049650	-0.204696

C	2.033140	-3.657702	1.589674
H	2.318009	-1.703274	-1.161689
H	2.461507	-4.548676	2.030156
C	0.931728	-1.351519	0.446586
C	1.024058	-2.955242	2.229479
O	0.535291	-3.363686	3.437087
C	0.471192	-1.815137	1.677084
H	-0.308743	-1.301605	2.219989
O	3.476232	-3.897682	-0.243039
H	3.731936	-3.482561	-1.076667
H	1.001353	-4.150270	3.748784
H	-3.546557	4.149471	0.195206
O	-1.153369	6.828530	-3.857506
H	-1.721962	6.311084	-3.264832
H	-1.359728	6.510685	-4.742859
O	0.076973	-1.205401	5.281901
H	0.233936	-1.961156	4.693100
H	0.506493	-0.459800	4.849120
O	4.850438	-6.076959	1.020629
H	4.411643	-5.337911	0.570110
H	5.220029	-5.694881	1.823780

- **S_1**

O	-2.362483	5.444755	-1.809582
H	-3.065673	5.849045	-1.282217
C	0.413370	-0.203781	-0.223413
H	0.870579	0.086104	-1.160838
C	-0.576080	0.641331	0.333650
H	-1.046670	0.347311	1.262829
C	-1.007600	1.841616	-0.222273
C	-0.488201	2.384040	-1.444370
H	0.282108	1.853596	-1.983972
H	-0.553738	3.975268	-2.868624
C	-0.949490	3.570320	-1.946374
C	-1.945370	4.279755	-1.271864
C	-2.481075	3.784913	-0.072757
C	-2.025218	2.604479	0.433986
H	-2.437695	2.222500	1.359256
C	2.317651	-3.335169	0.200161
C	1.866412	-2.161596	-0.335373
C	1.822496	-3.829495	1.413915
H	2.269272	-1.801877	-1.274456
H	2.197886	-4.758800	1.822318
C	0.860322	-1.399338	0.335654
C	0.843218	-3.092991	2.074575
O	0.327951	-3.530977	3.262804
C	0.357306	-1.910142	1.572792

H	-0.401444	-1.381550	2.129510
O	3.275456	-4.096384	-0.409838
H	3.578396	-3.669318	-1.221893
H	0.753050	-4.353485	3.537568
H	-3.252327	4.347991	0.438463
O	-1.622818	6.375440	-4.465284
H	-1.894035	6.082557	-3.582405
H	-1.880686	5.658645	-5.054887
O	-0.021309	-1.419640	5.178641
H	0.103718	-2.162602	4.565885
H	0.377065	-0.663068	4.735119
O	5.262553	-5.162150	1.367598
H	4.617027	-4.796003	0.741272
H	5.164145	-4.630869	2.164931

- **T_1**

O	6.374193	-0.042342	-0.145290
H	6.837985	0.805256	-0.150216
C	-0.125377	-0.502330	0.014912
H	0.238239	-1.514773	0.122646
C	0.842936	0.575669	-0.027408
H	0.482151	1.594713	-0.033569
C	2.222738	0.397277	-0.054782
C	2.857807	-0.881558	-0.054839
H	2.260389	-1.780534	-0.033241
H	4.693142	-1.975611	-0.081960
C	4.223130	-1.000985	-0.085711
C	5.026306	0.135652	-0.115377
C	4.444854	1.409428	-0.115730
C	3.085277	1.534564	-0.086925
H	2.638431	2.520487	-0.087329
C	-3.705466	-1.366047	-0.072702
C	-2.345265	-1.495613	0.001174
C	-4.313227	-0.115375	-0.215190
H	-1.895293	-2.474519	0.109583
H	-5.390447	-0.035621	-0.274816
C	-1.510486	-0.345225	-0.063855
C	-3.503282	1.010725	-0.281401
O	-4.046278	2.254532	-0.424009
C	-2.131510	0.922588	-0.208557
H	-1.548765	1.829105	-0.261085
O	-4.548587	-2.439591	-0.012250
H	-4.049636	-3.261474	0.081977
H	-5.010189	2.212454	-0.448370
H	5.080576	2.286508	-0.138800
O	7.478597	-2.202735	1.401601
H	7.145035	-1.470777	0.858936
H	7.477225	-2.968300	0.817312

O	-2.520777	4.657775	-0.136938
H	-3.060207	3.857800	-0.242634
H	-1.997743	4.714330	-0.943613
O	-6.708171	-2.422063	-1.893445
H	-5.993027	-2.465420	-1.237844
H	-7.422170	-1.948341	-1.453985

Cis-resveratrol

- **S_0**

O	-2.960738	3.834560	0.220151
H	-2.731862	4.033922	1.137295
C	1.342967	-0.732415	-1.694370
H	2.049488	-1.246656	-2.337112
C	0.688075	0.306843	-2.210265
H	0.856463	0.519179	-3.261155
C	-0.241223	1.228204	-1.536959
C	-0.055318	1.658433	-0.224844
H	0.802654	1.316514	0.336211
H	-0.789230	2.850682	1.400230
C	-0.946998	2.523679	0.379392
C	-2.046675	2.981800	-0.330278
C	-2.241149	2.589825	-1.644588
C	-1.336678	1.728716	-2.237165
H	-1.489437	1.429987	-3.266850
C	2.269958	-2.074749	1.677119
C	2.374429	-1.591669	0.382099
C	1.037083	-2.267284	2.273866
H	3.353573	-1.441846	-0.052492
H	0.954837	-2.641584	3.286266
C	1.224313	-1.277814	-0.329521
C	-0.102568	-1.979606	1.539361
O	-1.304240	-2.166088	2.163668
C	-0.023147	-1.494953	0.248300
H	-0.928992	-1.267871	-0.297440
O	3.432720	-2.347114	2.337345
H	3.254369	-2.681117	3.225984
H	-2.027746	-2.046237	1.533901
H	-3.095559	2.963802	-2.192609
O	-5.666401	3.012678	-0.245161
H	-4.771680	3.357736	-0.092245
H	-5.909490	3.324558	-1.123253
O	-1.413562	0.361864	3.643669
H	-0.699493	0.827343	3.194603
H	-1.425608	-0.521123	3.243587
O	5.400729	-0.272114	2.097811
H	4.770985	-1.003338	2.204674

H	5.922501	-0.502366	1.321784

- **S_1**

O	-3.082005	3.806609	0.109206
H	-2.894270	3.998773	1.038861
C	1.501143	-0.535424	-1.544285
H	2.437405	-0.664638	-2.080865
C	0.485988	0.220932	-2.184182
H	0.417779	0.149204	-3.266798
C	-0.381032	1.124942	-1.571612
C	-0.241718	1.543968	-0.208292
H	0.600359	1.198636	0.369639
H	-1.012954	2.745772	1.384245
C	-1.129142	2.429973	0.354020
C	-2.179202	2.938645	-0.400575
C	-2.331498	2.577537	-1.749526
C	-1.452532	1.705580	-2.317113
H	-1.569930	1.421815	-3.355310
C	2.430827	-2.300254	1.562816
C	2.537718	-1.736218	0.321983
C	1.204756	-2.380506	2.236987
H	3.505848	-1.664187	-0.155733
H	1.135966	-2.820830	3.223373
C	1.387258	-1.189202	-0.316430
C	0.070784	-1.903592	1.596893
O	-1.112422	-2.016014	2.276569
C	0.128340	-1.329330	0.347305
H	-0.784337	-1.029719	-0.145984
O	3.571974	-2.776767	2.148595
H	3.374962	-3.155090	3.015155
H	-1.847727	-1.768244	1.699904
H	-3.150787	2.999102	-2.316372
O	-5.869448	3.290182	-0.507072
H	-4.949319	3.514174	-0.299151
H	-6.047760	3.720132	-1.350282
O	-0.992077	0.456585	3.838366
H	-0.393588	0.968320	3.283058
H	-1.058248	-0.407238	3.402950
O	5.434329	-0.588534	2.259667
H	4.851985	-1.365164	2.234389
H	4.929922	0.114451	1.836609

- **T_1**

O	-3.062484	4.245190	-0.433602
H	-2.794222	4.617075	0.416771
C	1.435217	-0.415385	-1.444096
H	2.419872	-0.359965	-1.899769

C	0.314392	0.158147	-2.170929
H	0.097927	-0.244593	-3.155660
C	-0.498166	1.216072	-1.715764
C	-0.255728	1.903927	-0.505370
H	0.600599	1.634065	0.095878
H	-0.890190	3.425093	0.859931
C	-1.089666	2.910600	-0.072503
C	-2.198873	3.265981	-0.827753
C	-2.461737	2.620848	-2.033692
C	-1.624927	1.624559	-2.468842
H	-1.834741	1.122602	-3.404793
C	2.459092	-2.158819	1.651550
C	2.527163	-1.540407	0.423307
C	1.245502	-2.348083	2.301068
H	3.486940	-1.394460	-0.053411
H	1.198855	-2.823784	3.272265
C	1.349324	-1.083063	-0.202264
C	0.083955	-1.911284	1.676544
O	-1.087386	-2.128589	2.343016
C	0.114039	-1.291011	0.447393
H	-0.807326	-0.962289	-0.012622
O	3.631194	-2.577410	2.213914
H	3.474422	-2.966180	3.084057
H	-1.831319	-1.764145	1.845705
H	-3.329512	2.911395	-2.610922
O	-5.862512	3.755458	-0.794794
H	-4.921199	3.957937	-0.669872
H	-6.103202	4.180892	-1.624526
O	-0.994793	-1.644202	5.168115
H	-0.289664	-0.996763	5.274308
H	-1.061212	-1.789887	4.210428
O	5.751363	-0.672045	1.871668
H	5.055872	-1.328816	2.037668
H	5.280481	0.145718	1.678606

THP

- **S_0**

O	4.063477	-1.138546	1.366062
H	5.002833	-0.957678	1.140268
C	-0.829288	1.347060	-1.864494
H	-1.513044	1.862257	-2.526885
C	0.499980	1.466440	-2.016647
H	0.913654	2.081528	-2.806605
C	1.404634	0.789706	-1.144807
C	0.913918	-0.027204	-0.095008
H	1.552909	-1.287601	1.539000

C	1.865582	-0.658381	0.726573
C	3.218725	-0.495458	0.525382
C	3.694028	0.310526	-0.516096
C	2.787307	0.936067	-1.327944
H	3.134752	1.565091	-2.138434
C	-3.358709	-0.317484	0.255827
C	-2.782029	0.453100	-0.724909
C	-2.546010	-1.015019	1.148283
H	-3.396683	0.999249	-1.427749
H	-3.003333	-1.622519	1.917575
C	-1.386310	0.534377	-0.824121
C	-1.172079	-0.944327	1.063701
O	-0.427116	-1.637916	1.949420
C	-0.529094	-0.159314	0.066106
O	-4.694572	-0.441696	0.409754
H	-5.180058	0.081553	-0.267901
H	-0.994319	-2.131662	2.585031
H	4.758221	0.432405	-0.668127
O	6.717498	-0.757700	0.933205
H	7.042231	-1.001128	0.057470
H	7.022052	0.146570	1.078599
O	-1.867178	-3.038290	3.768804
H	-1.551836	-3.944325	3.874679
H	-1.832945	-2.646220	4.650105
O	-6.092167	0.952542	-1.454776
H	-6.288276	1.860545	-1.192906
H	-6.944746	0.553330	-1.667508

- **S_1**

O	4.051425	-1.170094	1.339032
H	5.000684	-0.998877	1.131335
C	-0.851378	1.371615	-1.841764
H	-1.525651	1.899676	-2.504365
C	0.529161	1.497143	-2.009333
H	0.929509	2.119366	-2.798399
C	1.414517	0.821917	-1.163366
C	0.908457	-0.026376	-0.087882
H	1.556308	-1.302104	1.520001
C	1.859162	-0.664270	0.711059
C	3.229508	-0.508064	0.505610
C	3.717834	0.304694	-0.526555
C	2.815969	0.951698	-1.337772
H	3.169604	1.585096	-2.141314
C	-3.372442	-0.297492	0.284764
C	-2.795902	0.482551	-0.705206
C	-2.563949	-1.002890	1.173851
H	-3.427389	1.029249	-1.393735

H	-3.009110	-1.614276	1.945213
C	-1.391114	0.578839	-0.836496
C	-1.169896	-0.924415	1.064411
O	-0.437665	-1.619898	1.939593
C	-0.522139	-0.145932	0.073812
O	-4.705461	-0.407945	0.431554
H	-5.188572	0.112222	-0.253245
H	-1.001969	-2.114105	2.583788
H	4.783932	0.414838	-0.673408
O	6.695698	-0.822370	0.950777
H	7.025569	-1.061036	0.075483
H	7.004979	0.078773	1.106375
O	-1.836717	-3.008990	3.762718
H	-1.514841	-3.913126	3.866382
H	-1.796150	-2.614638	4.642858
O	-6.079406	0.972503	-1.439917
H	-6.581200	1.719749	-1.091327
H	-6.719352	0.426847	-1.913601

- **T_1**

O	4.056188	-1.083159	1.281168
H	4.999800	-0.894681	1.067447
C	-0.956906	1.375346	-1.808403
H	-1.648236	1.892771	-2.460022
C	0.455499	1.533448	-2.002671
H	0.821595	2.167721	-2.797691
C	1.350157	0.878466	-1.179726
C	0.886497	0.023441	-0.108266
H	1.553854	-1.244491	1.478653
C	1.841171	-0.598088	0.669558
C	3.223547	-0.421986	0.452193
C	3.677257	0.399445	-0.576433
C	2.757521	1.032330	-1.370216
H	3.089831	1.675126	-2.175179
C	-3.410708	-0.338231	0.342799
C	-2.867550	0.453190	-0.652383
C	-2.570849	-1.019842	1.203067
H	-3.509957	0.993514	-1.334820
H	-2.985476	-1.643695	1.982381
C	-1.460474	0.569437	-0.797048
C	-1.166216	-0.914252	1.072758
O	-0.411846	-1.607587	1.950194
C	-0.569935	-0.132668	0.087876
O	-4.743566	-0.476582	0.515722
H	-5.245192	0.034696	-0.158970
H	-0.967334	-2.108514	2.590538

H	4.738710	0.532795	-0.738414
O	6.664271	-0.565523	0.771852
H	7.057885	-1.155960	0.117520
H	7.222440	-0.637401	1.556001
O	-1.804324	-3.081180	3.742594
H	-1.407548	-3.951941	3.869022
H	-1.855158	-2.688102	4.622640
O	-6.280858	0.846172	-1.285090
H	-6.198432	0.508119	-2.185427
H	-6.133648	1.797746	-1.352299

- **T_2**

O	4.043896	-1.264911	1.293374
H	4.984743	-1.080753	1.076148
C	-0.851051	1.441144	-1.819680
H	-1.531185	1.969918	-2.476465
C	0.494757	1.581749	-1.961312
H	0.909519	2.223754	-2.725974
C	1.402209	0.865429	-1.112281
C	0.884453	-0.014433	-0.071703
H	1.542420	-1.395966	1.471954
C	1.851363	-0.715664	0.701957
C	3.207118	-0.554593	0.501999
C	3.693230	0.307240	-0.482619
C	2.770912	1.000743	-1.277194
H	3.133339	1.663821	-2.051604
C	-3.369467	-0.321851	0.256595
C	-2.784352	0.480568	-0.724704
C	-2.561940	-1.006256	1.182557
H	-3.419612	1.013243	-1.421451
H	-3.022292	-1.601885	1.956846
C	-1.404981	0.609996	-0.822037
C	-1.191536	-0.901567	1.113926
O	-0.449004	-1.529108	2.019909
C	-0.518180	-0.115352	0.093138
O	-4.693055	-0.463319	0.368405
H	-5.177520	0.053604	-0.323507
H	-1.000886	-2.031176	2.672906
H	4.757691	0.427589	-0.628812
O	6.704223	-0.902802	0.850695
H	7.009225	-1.115107	-0.040125
H	7.027762	-0.010621	1.026769
O	-1.805048	-2.961917	3.820056
H	-1.429463	-3.846293	3.914991
H	-1.794077	-2.577151	4.705433
O	-6.025739	0.900888	-1.514550
H	-6.522577	1.659507	-1.183558

H	-6.662888	0.361411	-1.999153

- **T_3**

O	4.025217	-1.198818	1.367918
H	4.967746	-1.030202	1.144698
C	-0.849290	1.408209	-1.858960
H	-1.527102	1.929747	-2.524045
C	0.497718	1.531355	-2.009522
H	0.917027	2.150160	-2.791331
C	1.399719	0.835448	-1.137481
C	0.874728	-0.011442	-0.070966
H	1.524243	-1.321551	1.537562
C	1.837554	-0.676519	0.739642
C	3.194091	-0.524424	0.540495
C	3.686682	0.294319	-0.477062
C	2.768875	0.960378	-1.301653
H	3.136702	1.595233	-2.097376
C	-3.380707	-0.274170	0.267416
C	-2.788094	0.504590	-0.728669
C	-2.578996	-0.973761	1.185847
H	-3.420433	1.040125	-1.425256
H	-3.042803	-1.567185	1.959759
C	-1.408511	0.605720	-0.841817
C	-1.207678	-0.897269	1.102672
O	-0.472164	-1.557536	1.989580
C	-0.527031	-0.115214	0.082562
O	-4.704760	-0.384354	0.394782
H	-5.188886	0.138124	-0.293533
H	-1.031635	-2.054376	2.640267
H	4.752175	0.405841	-0.622591
O	6.685286	-0.877929	0.915815
H	6.984771	-1.159769	0.042761
H	7.013501	0.023490	1.021798
O	-1.862659	-2.946300	3.798108
H	-1.529535	-3.846441	3.901384
H	-1.836739	-2.554276	4.679860
O	-6.053403	1.007882	-1.448649
H	-6.601410	1.711011	-1.078365
H	-6.647285	0.467612	-1.984476

Resveratrone

- **S_0**

H	-0.989308	1.303595	-3.906706
C	-1.349026	1.260785	-2.887877
C	-2.570722	1.777274	-2.563004
C	-3.067209	1.742468	-1.247212

C	-2.317266	1.177904	-0.251249
C	-1.033663	0.625282	-0.541431
C	-0.561164	0.675692	-1.878022
C	0.712683	0.123881	-2.151187
C	1.459973	-0.437237	-1.161586
C	0.992930	-0.489315	0.174735
C	-0.244301	0.042590	0.459618
H	-4.040562	2.161603	-1.025702
H	-0.633054	0.021611	1.467055
H	2.432949	-0.854692	-1.389378
H	1.085366	0.155874	-3.167013
C	1.846822	-1.102223	1.182102
H	2.793646	-1.471019	0.803054
C	1.583962	-1.247146	2.485483
H	0.656390	-0.900185	2.923721
C	2.502338	-1.870985	3.431383
C	3.829192	-2.396780	2.967416
H	4.362156	-2.834024	3.806717
H	3.690370	-3.151535	2.193043
H	4.426669	-1.592919	2.536443
O	-3.318519	2.336945	-3.555471
H	-4.144480	2.695231	-3.205804
O	-2.732998	1.113752	1.022444
H	-3.632159	1.508350	1.130903
O	2.174549	-1.957453	4.611413
O	3.809143	-2.985324	6.610967
H	4.612284	-2.454477	6.602106
H	3.252984	-2.620300	5.894338
O	-1.840990	3.794322	-5.538655
H	-1.025676	4.036721	-5.086794
H	-2.369444	3.329212	-4.870253
O	-5.191781	2.141794	1.334087
H	-5.237913	2.898965	1.930995
H	-5.832511	1.503191	1.670891

- **S_1**

H	-1.008567	1.357646	-3.844600
C	-1.350913	1.284898	-2.821233
C	-2.613930	1.802960	-2.486074
C	-3.097157	1.739604	-1.191540
C	-2.323858	1.154466	-0.201817
C	-1.020612	0.609042	-0.502982
C	-0.563757	0.697692	-1.853333
C	0.729122	0.159159	-2.157926
C	1.491076	-0.413678	-1.196656
C	1.052289	-0.509883	0.155146
C	-0.241038	0.026364	0.465953

H	-4.071170	2.143393	-0.947969
H	-0.614218	-0.024494	1.476547
H	2.465241	-0.814477	-1.446798
H	1.083208	0.221755	-3.178593
C	1.883520	-1.110762	1.113623
H	2.836577	-1.474286	0.750621
C	1.579104	-1.264785	2.462105
H	0.633482	-0.906547	2.850499
C	2.432519	-1.863885	3.424389
C	3.779488	-2.395183	3.008047
H	4.239981	-2.923522	3.838893
H	3.695438	-3.072289	2.157133
H	4.436835	-1.577529	2.703659
O	-3.321636	2.356573	-3.487775
H	-4.170751	2.701461	-3.177015
O	-2.725966	1.065209	1.047730
H	-3.632235	1.464863	1.193335
O	2.084437	-1.949459	4.630414
O	3.675216	-2.899436	6.589053
H	4.511144	-2.426166	6.532241
H	3.124349	-2.548191	5.848432
O	-1.962348	3.671060	-5.716696
H	-1.189340	4.073035	-5.305877
H	-2.438398	3.245015	-4.988836
O	-5.094946	2.112180	1.436596
H	-5.096357	2.873818	2.030277
H	-5.745792	1.494833	1.794072

- **T_1**

H	-1.014886	1.270378	-3.908839
C	-1.360911	1.210816	-2.885320
C	-2.602678	1.752326	-2.546299
C	-3.083699	1.699512	-1.246833
C	-2.320714	1.102736	-0.259052
C	-1.042706	0.540461	-0.563153
C	-0.588758	0.612988	-1.912610
C	0.700711	0.042512	-2.219228
C	1.462766	-0.528740	-1.267548
C	1.033958	-0.602461	0.100997
C	-0.257123	-0.053124	0.412561
H	-4.048777	2.125227	-1.002893
H	-0.627203	-0.089773	1.423645
H	2.429536	-0.945728	-1.519916
H	1.045742	0.089806	-3.244559
C	1.871876	-1.173467	1.049406
H	2.825654	-1.541137	0.695849
C	1.573594	-1.290887	2.419179

H	0.624062	-0.944390	2.804236
C	2.454957	-1.833454	3.399584
C	3.807023	-2.349960	2.993354
H	4.321763	-2.758164	3.858580
H	3.711842	-3.126040	2.232890
H	4.409217	-1.548559	2.562598
O	-3.313888	2.331137	-3.545664
H	-4.146434	2.694669	-3.214517
O	-2.728702	1.023961	1.007707
H	-3.616287	1.443891	1.142624
O	2.100914	-1.875769	4.595024
O	3.713440	-2.759595	6.630910
H	4.519114	-2.234184	6.594379
H	3.164605	-2.444386	5.880585
O	-1.717872	3.966737	-5.328923
H	-0.956052	4.204875	-4.789575
H	-2.291139	3.452817	-4.739282
O	-5.102136	2.159833	1.414933
H	-5.078582	2.884734	2.052218
H	-5.780567	1.551834	1.734520

- **T_2**

H	-1.044926	1.354690	-3.877216
C	-1.386729	1.266996	-2.855172
C	-2.635118	1.772503	-2.499874
C	-3.117227	1.690584	-1.211480
C	-2.313666	1.078765	-0.213992
C	-1.035708	0.555275	-0.523062
C	-0.562597	0.642290	-1.871530
C	0.686200	0.118480	-2.176362
C	1.487049	-0.477455	-1.194704
C	1.035371	-0.555726	0.128342
C	-0.227040	-0.038821	0.444662
H	-4.090698	2.086450	-0.956401
H	-0.595990	-0.089651	1.457556
H	2.458564	-0.874481	-1.452596
H	1.044757	0.180869	-3.195993
C	1.880338	-1.156621	1.130739
H	2.843672	-1.502297	0.776172
C	1.581245	-1.318377	2.463061
H	0.629863	-0.994700	2.865270
C	2.476959	-1.895186	3.432148
C	3.830057	-2.391111	3.012333
H	4.340806	-2.830258	3.864520
H	3.740297	-3.136994	2.221785
H	4.430515	-1.570472	2.616788
O	-3.358364	2.350393	-3.493357

H	-4.194922	2.697023	-3.154830
O	-2.740774	0.984462	1.046566
H	-3.625974	1.409837	1.179171
O	2.116721	-1.971683	4.616466
O	3.732925	-2.922086	6.648976
H	4.539883	-2.398008	6.619038
H	3.181965	-2.587926	5.911532
O	-1.832047	3.986374	-5.323065
H	-1.070111	4.259110	-4.800613
H	-2.375825	3.459886	-4.716267
O	-5.084789	2.098445	1.616309
H	-5.860116	1.761898	1.149782
H	-5.121775	3.058991	1.527224
